# Editorial: Contribution of Translational Animal Models to the Systems Biology of Neurodegenerative Disorders

**DOI:** 10.3389/fphys.2020.00775

**Published:** 2020-07-24

**Authors:** Wael M. Y. Mohamed, Mohamed M. Salama, Maha M. El Batsh, Eyleen L. Goh

**Affiliations:** ^1^Basic Medical Science Department, International Islamic University Malaysia, Selayang, Malaysia; ^2^Institute of Global Health and Human Ecology, The American University in Cairo, Cairo, Egypt; ^3^Clinical Pharmacology Department, Menoufia University, Shebeen El-Kom, Egypt; ^4^Department of Research, National Neuroscience Institute, Singapore, Singapore

**Keywords:** system biology, animal models, neurodegenerative disorders, translational research, complex pathophysiology, biomarkers

Neurodegenerative disorders (NDs) are the second leading causes of death and the leading causes of disability worldwide causing significant health and socioeconomic problems. The burdens of NDs on healthcare systems continue to increase despite more than 25 years of implementing various healthcare policies and intervention programs worldwide. Population growth and increased aging population likely contributed to the continuous rise in incidence, prevalence, mortality, and impairment rates of NDs (GBD 2016 Neurology Collaborators, 2019). This global and continuous increase in NDs suggests an urgent need to find new approaches to improve prognosis, enable early diagnosis, increase efficiency of drug discovery, establish a more personalized medicine approach as well as improve patient care and manage rising health costs.

NDs are generally complex multigenic disorders involving multiple genes, cell types, and brain regions. The underlying etiologies of most NDs are still largely unknown. Furthermore, there is a lack of clear understanding on the underlying pathophysiological mechanisms and their dynamic interactions at cellular level leading to NDs. Limited understanding on mechanisms of NDs is the bottleneck that hinders effective disease detection and management, thus explains the current lack of effective treatments for NDs. To better understand the complex pathophysiology of NDs and drug development, modeling of physiological processes in these human diseases through system biology at the cellular, molecular, and genetic levels is important. This is because conventional approaches to medical and biological interpretations are only appropriate for processing with a small number of components and short causal chains. However, broad ranges of components that communicate across elaborated networks are known to be the underlying causes of many complex diseases in humans. New and more sophisticated ways of fueling further developments in complex diseases are therefore needed (Wolkenhauer et al., 2009).

System biology is a systematic analysis of complex and diverse molecular and regulatory networks that eventually decode and classify individual living systems. Nonetheless, it is worth noting that classifying casual pathways and pathophysiology in NDs is not possible by using terminal human tissues alone. Moreover, performing system biology experiments in humans is not feasible. Thus, animal models such as rat, mouse, nematode, and fruit fly are commonly used to investigate the mechanistic connections to neurodegenerative processes and to search for potential therapeutics. Similar neurological and neuropathological analogies of neurodegeneration in human subjects can typically be at least partially recapitulated in these non-human disease models. The evolutionary conserved genetic, molecular, and cellular modifications that underlie brain functions in humans and other species will help to understand the underlying pathophysiological changes in human disorders.

This volume aimed to provide an overview of contributions from structural, empirical, and computational modeling of the different translational *in vivo* animal systems to understand NDs. The authors from different research fields identified numerous existing and emerging animal models to research on neurodegeneration that provide new and unique perspectives. In this volume, several articles focus on comparing different animal models for Alzheimer's disease (AD) and other NDs. Mustapha et al., integrated contemporary findings from various experimental animal models of cerebral small vessel disease (CSVD) to date, and detailed their translational importance for science. The authors also highlighted different system biology options for CSVD and further described pathomechanical aspects of blood brain barrier (BBB) damage in relation to CSVD, as well as biomarkers for early disease diagnosis and converging strategies for future therapeutic directions of CSVD.

In another review, Hoffe and Holahan presented a systematic distinction between pigs and humans with other animal models for NDs. The authors advocated the use of pigs as a translational model for studying NDs. There are many similarities between porcine and human brains. Longer life spans in pigs allow longitudinal studies to assess the long-term consequences and natural development of NDs. However, the lack of sophisticated behavioral knowledge within literature suggests that more research is required to improve the viability of using pigs to study NDs.

Using different animal models for transthyretin amyloidosis (ATTR), a lethal systemic disease caused by misfolded transthyretin deposits, Ibrahim et al., identified the breakthrough, progress and difficulties in developing animal models for research into ATTR. Models of ATTR were generated using fruit flies, rats, mice, non-human primates and induced pluripotent stem cells. Since none of these models of ATTR can fully recapitulate all of the disease's symptoms, it will be beneficial to combine multiple animal models to accurately mimic and evaluate different clinical features/symptoms of ATTR. Therefore, these animal models played a major role in the understanding of disease pathology, biomarker discovery, and possible therapeutic targets.

Over the years, several different drug targets for NDs have been suggested and experimented, especially those that display anti-inflammatory and antioxidant effects. Here, Bhuvanendran et al. demonstrated embeline (2,5- dihydroxy-3-undecyl-1,4-benzoquinone), a class of benzoquinone naturally contained in bright orange fruits, as a potential therapeutic molecule for AD. This is mainly because embeline exhibits anti-inflammatory, antioxidant, analgesic, antifertility, antitumor, wound healing, hepatoprotective and antibacterial activities. Bhuvanendran et al., showed that embelin is a strong inhibitor of acetylcholinesterase (AChE) in cells and could cross the BBB. These data were further confirmed by the findings *in silico*. Moreover, molecular docking experiments predicted that embelin has favorable binding mode within the active sites of AChE and amyloid beta (Aβ) peptide. Thus, this study identified embeline as a favorable compound which can be further developed into a potential multipotent therapeutic agent for AD.

Another research article by Jin et al. proposed that proteostasis network targeting protein misfolding may be dysfunctional in NDs. Binding immunoglobulin protein (BiP), an endoplasmic reticulum chaperone essential for protein folding and adaptive response modulation in early secretory pathways, interacts with unfolded proteins via its substratum-binding domain and nucleotide-binding domain with ATPase activity. This study suggested that decreased regulation of protein quality promotes cognitive dysfunction associated with aging. Impairment of the proteostasis network can thus facilitate age-related neurodegeneration, and this is aggravated by external environmental insults.

Overall, this volume provides a rare opportunity to promote awareness and creativity for neurological disorders using animal models of different species, while stimulating testable theories and establishing a strategic research agenda to facilitate their integration into clinical studies.

## Author Contributions

WM wrote the original draft and incorporated suggestions from the co-authors. The other co-authors contributed to the study and edited the manuscript.

## Conflict of Interest

The authors declare that the research was conducted in the absence of any commercial or financial relationships that could be construed as a potential conflict of interest.

## References

[B1] GBD 2016 Neurology Collaborators (2019). Global, regional, and national burden of neurological disorders, 1990-2016: a systematic analysis for the Global Burden of Disease Study 2016. Lancet Neurol. 18, 459–480. 10.1016/S1474-4422(18)30499-X 30879893 PMC6459001

[B2] Wolkenhauer O. Fell D. De Meyts P. Blüthgen N. Herzel H. Le Novère N.. (2009). SysBioMed report: advancing systems biology for medical applications. IET Syst. Biol. 3, 131–136. 10.1049/iet-syb.2009.0005 19449974

